# A Comprehensive Approach to the Diagnosis of Leigh Syndrome Spectrum

**DOI:** 10.3390/diagnostics14192133

**Published:** 2024-09-25

**Authors:** Manuela Schubert Baldo, Luísa Azevedo, Margarida Paiva Coelho, Esmeralda Martins, Laura Vilarinho

**Affiliations:** 1 Research and Development Unit, Human Genetics Department, National Institute of Health Doutor Ricardo Jorge, 4000-053 Porto, Portugal; 2 School of Medicine and Biomedical Sciences (ICBAS), University of Porto, 4050-313 Porto, Portugal; mmargaridacoelho.dca@chporto.min-saude.pt (M.P.C.); esmeralda.dia@chporto.min-saude.pt (E.M.); 3 UMIB—Unit for Multidisciplinary Research in Biomedicine, School of Medicine and Biomedical Sciences (ICBAS), University of Porto, 4050-346 Porto, Portugal; lazevedo@icbas.up.pt; 4 ITR—Laboratory for Integrative and Translational Research in Population Health, 4050-600 Porto, Portugal; 5 Pediatrics Department, Northern Mother and Child Centre, Reference Centre for Metabolic Disorders, Santo António Hospital University Centre, 4050-651 Porto, Portugal; 6 Neonatal Screening, Metabolism and Genetics Unit, Human Genetics Department, National Institute of Health Doutor Ricardo Jorge, 4000-053 Porto, Portugal

**Keywords:** Leigh syndrome spectrum, diagnosis, mitochondrial disorder, neurodegeneration

## Abstract

Background: Leigh syndrome spectrum (LSS) is a novel nomenclature that encompasses both classical Leigh syndrome and Leigh-like phenotypes. Given the heterogeneity of disease presentation, a new consensus published recently addressed the main issues and proposed general guidelines towards diagnosis. Based on these recommendations, we developed a simple pipeline that can be useful in the diagnosis of LSS. Methods: We combined previously published criteria with our own experience to achieve a diagnostic framework that can provide faster satisfactory results with fewer resources. Results: We suggest adding basic biochemical tests for amino acids, acylcarnitine, and urinary organic acids as parallel investigations, as these results can be obtained in a short time. This approach characterized 80% of our cohort and promoted specific intervention in 10% of confirmed cases. Conclusions: Genetic studies are crucial in the diagnosis of LSS, but they are time-consuming and might delay tailored interventions. Therefore, we suggest adding more affordable and less complex biochemical studies as primary tests when investigating treatable causes of LSS.

## 1. Introduction

Leigh syndrome (LS) is the most frequent mitochondrial neurodegenerative disorder in childhood, affecting approximately 1:40.000 live births [1,2,3] with predominantly neurologic involvement, such as neurodevelopmental delay, regression, or psychiatric symptoms [4,5,6]. The first case was described by Denis Archibald Leigh in 1951 and revealed a constellation of isolated neurological findings in the autopsy of a 7-month-old boy [7]. Currently, the term “spectrum” is a new designation merging Leigh and Leigh-like syndrome that highlights the disease as a continuum and includes suspicious cases that exhibit atypical presentations in the Leigh syndrome “universe” [5,8]. Symptoms primarily result from the dysfunction of the central nervous system and manifest as epilepsy, motor and/or speech delay, hypotonia, apnea, pyramidal signs, and movement disorders [4]. These clinical manifestations correspond to neuroimaging features of bilateral symmetric basal ganglia involvement as well as brainstem and spinal cord regions. Typically, the first symptoms begin in the first years of life, but there is an increasingly number of cases with adulthood presentation [9]. Atypical and/or mild presentations have been observed in adulthood mimicking Parkinson disease, spinocerebellar ataxia, and multiple sclerosis [10].

An intact oxidative phosphorylation (OXPHOS) system is essential to the aerobic metabolism. When hampered, glycolytic products such as lactate (L) and/or pyruvate (P) are abnormally high, demonstrating an ineffective aerobic metabolism. Lactate is an intermediate compound of energy production, considered a noble part in the redox signaling state and a regulator of homeostasis, representing a relevant source of energy [11]. Lactate and pyruvate can accumulate in body fluids such as blood, urine, and cerebral spinal fluid (CSF). Double lactate peaks or a pyruvate peak in magnetic resonance spectroscopy (MRS) in correspondent topography for LSS is valued as biochemically supportive of the diagnosis [5]. Because pyruvate has a variable range of sensitivity and a strong specificity threshold, it is usually considered in association with lactate, using the ratio between them (an L/P ratio higher than 20) as a more specific biomarker for mitochondrial disorders (MDs) [12]. High alanine can also reflect a glycolytic metabolism through the conversion of lactate to alanine; therefore, it is an important marker in suspected MDs. OXPHOS functional studies (OXPHOS complex activities), usually performed in muscle biopsies, are closest to mitochondrial metabolism, as they are considered to “mirror” the patient’s metabolic condition [13]. Despite being highly recommended in clinical practice, deep tissue biopsy requires invasive procedures and anesthesia, adding risks for patients.

LSS may arise either from mitochondrial DNA (mtDNA) or nuclear DNA (nDNA) mutations via autosomal dominant, autosomal recessive, X-linked, or maternal inheritance. Identification of the inheritance mode is crucial for genetic counseling. *MT-ATPase6* m.8993T>G/C stands out as a frequent pathological variant in mtDNA marked by hypocitrullinemia in most cases, while in nuclear genome human surfeit 1 (*SURF1*), leucine rich pentatricopeptide repeat containing (*LRPPRC*) complex IV (COX) and complex I (CI) deficiencies are more frequently seen [3]. However, the absence of a straightforward genotype–phenotype relationship is a characteristic of many genetic diseases, such as LSS, and arises largely due to incomplete penetrance and variable expressivity [14,15]. The phenotype of mtDNA carriers linked to LSS is variable, which indicates that the associated genetic background and/or environmental variables can alter the expression and penetrance of disease-causing variants. Their interactions might result in a multitude of clinical presentations with varying degrees of severity, making it difficult to predict the outcome associated with a specific pathological variant [16,17,18].

LSS criteria were recently revisited by the Clinical Genome Resource (ClinGen) Mitochondrial Disease Gene Curation Expert Panel (Mito GCEP) [5]. A patient displaying a combination of neurologic symptoms and compatible neuroradiologic criteria supported by biochemical findings most likely has LSS. Neuroimaging criteria include bilateral, typically symmetric, T2-weighted hyperintensities on MRIs or hypodensities on CT scans of the brainstem and/or basal ganglia with or without bilateral T2 hyperintensity on MRIs or hypodensity on CT scans of the thalamus, cerebellum, subcortical white matter, and/or spinal cord [5]. The observation of neurological symptoms, such as delay, regression, or psychiatric symptoms, obtained with predefined biochemical tests and neuroimaging studies might not result in a definitive diagnosis. Current diagnostic strategies include mandatory genetic confirmation [6,8,19,20]. 

In high-income countries, whole exome sequencing (WES) is often a readily available first line test. However, in other economies, technical and financial constraints interfere with following such guidelines, turning the diagnostic process into an odyssey for patients and their families. Moreover, not all cases are solved through genetic studies, and inconclusive results are more frequent than desired. 

We propose a cost- and time-effective pipeline based on Mito GCEP work [5] and our own experience, prioritizing frequent and treatable causes, which can be adjusted to clinical practices of different realities.

## 2. Materials and Methods

We revisited a cohort of 50 LSS-suspected individuals (23 males and 27 females) investigated at our center during the last three decades. These patients were studied through a sequential gene-targeted approach for the most frequent mtDNA and nDNA mutations in diagnosed LSS cases [21,22]. We identified practical cases in which a more comprehensive initial approach than that proposed by the Mito GCEP would benefit a timelier diagnosis and phenotype–genotype correlation, enabling adaption to each service. Other causes of LSS with easily obtainable biochemical markers identified through a literature review from other groups were also included. Neuroimaging patterns and criteria were previously defined [5] and are outside the focus of this work.

## 3. Results

**Figure 1:**
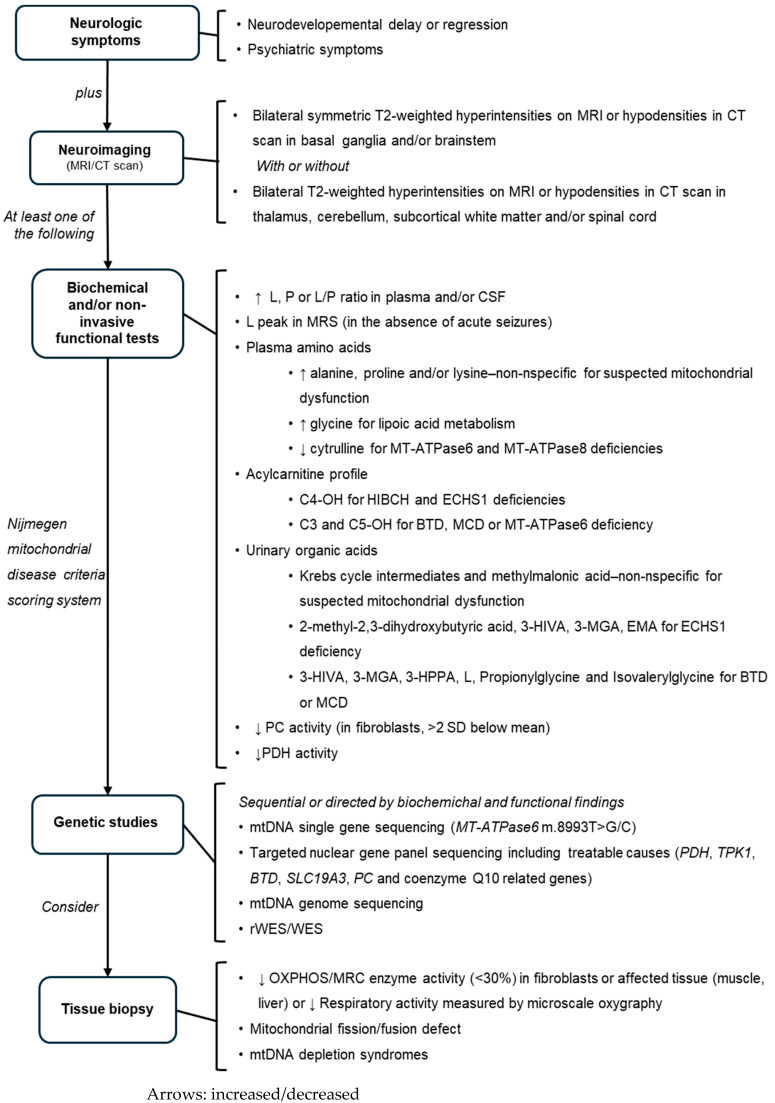
Proposed algorithm for the diagnosis of LSS. Abbreviations: L = lactate; P = pyruvate; CSF = cerebrospinal fluid; CT = computed tomography; LSS = Leigh syndrome spectrum; MRI = magnetic resonance imaging; MRS = magnetic resonance spectroscopy; C4-OH = 3-hydroxybutyrylcarnitine; C3 = propionylcarnitine; C5-OH = 3-hydroxyvalerylcarnitine; HIBCH = 3-hydroxyisobutyryl-CoA hydrolase; ECHS1 = short-chain enoyl-CoA hydratase deficiency; BTD = biotinidase deficiency; MCD = multiple carboxylase deficiency; 3-HIVA = 3-hydroxyisovaleric acid; 3-MGA = methylglutaconic acid; EMA = ethylmalonic acid; 3-HPPA = 3-hydroxy propionic acid; PC = pyruvate carboxylase; SD = standard deviation; PDH = pyruvate dehydrogenase; SLC19A3 = solute carrier family 19 member 3; rWES = rapid whole exome sequencing; WES = whole exome sequencing; OXPHOS = oxidative phosphorylation; MRC = mitochondrial respiratory chain.

Considering our expertise and the literature reviewed, we propose a diagnostic protocol for the diagnosis of LSS, focusing on treatable causes and shortening diagnostic time. Primarily based on the Mito GCEP’s recommendations, our approach can be adapted to distinct economies, as shown in Figure 1.

From a clinical perspective, the phenotype is still a relevant factor and can suggest a molecular diagnosis, such as *NDUFV2* mutations identified in two siblings of our cohort displaying encephalopathy and cardiomyopathy. However, MDs are such a heterogenic group of disorders that they require the more robust support given by the following steps of investigation [21,22]. To support our suggestions, we revisited data previously collected through phenotype characterization and basic biochemical studies leading to a genetic diagnosis in a few of our cases.

*MT-ATPase6* mutations, especially m.8993T>G/C, were the most frequently found variants (16/50). *MT-ATPase6* m.8993T>G/C and m.9176T>G/C impact complex V (CV) and result in low to absent citrulline levels in approximately 80% of patients [23,24,25,26]. Amino acid studies are also helpful in distinguishing MDs from pyruvate dehydrogenase deficiency (PDHD), a known cause of LSS in which branched chain amino acids follow plasma alanine levels. According to the Nijmegen mitochondrial disease criteria scoring system, alanine levels above 450 μM are a highly sensitive parameter for MDs [27,28].

Organic acidurias are also a known cause of LSS commonly detected through a urinary organic acids profile. Mitochondrial enzyme defects HIBCHD and ECHS1D are well established causes of LSS resulting from an impairment in valine metabolism [29,30,31]. They differ from each other biochemically by urinary and acylcarnitine profile and represent a treatable cause of LSS [29].

**Figure 2:**
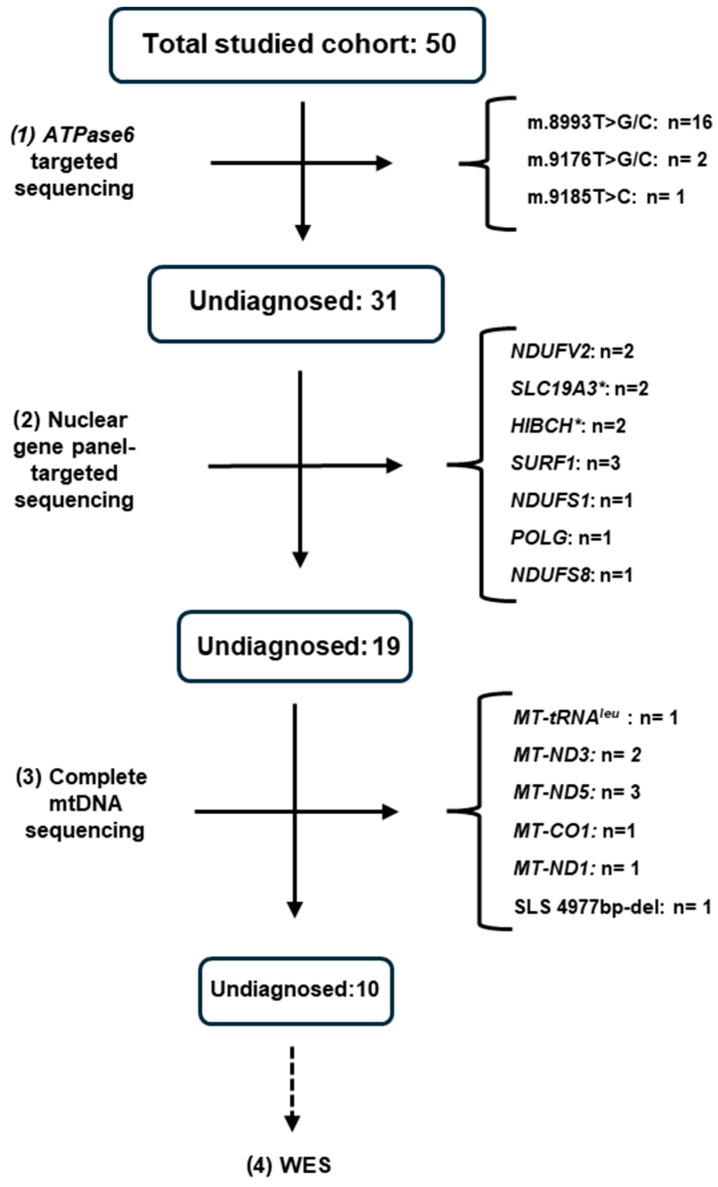
Results of molecular studies in our clinical cohort. * = treatable cases in our cohort; NDUFV2 = NADH:ubiquinone oxidoreductase core subunit v2; SLC19A3 = solute carrier family 19 member 3; HIBCH = 3-hydroxyisobutyryl-CoA hydrolase; SURF = human surfeit 1; NDUFS1 = NADH:ubiquinone oxidoreductase core subunit S1; POLG = DNA polymerase subunit gamma; NDUFS8 = NADH:ubiquinone dehydrogenase iron-sulfur protein 8; MT-tRNA^leu^ = mitochondrially encoded tRNA leucine 1; MT-ND3 = mitochondrially encoded NADH:ubiquinone oxidoreductase core subunit 3; MT-ND5 = mitochondrial genome coding for the NADH-ubiquinone oxidoreductase chain 5 protein; MT-CO1 = mitochondrially encoded cytochrome c oxidase 1; MT-ND1 = mitochondrially encoded NADH:ubiquinone oxidoreductase core subunit 1; SLS 4977bp-del = single-large scale deletion of 4977 bp; WES = whole exome sequencing.

In our experience, 10% of characterized cases (4/40) corresponded to treatable etiologies, with two cases bearing *SLC19A3* mutations and two with *HIBCH* mutations [21]. An *HIBCH* patient presented failure to thrive in early months of life. Urinary organic acids displayed valine pathway metabolites, which led us to first consider ECSH1D, another treatable cause of LSS. However, acylcarnitine profile showed high levels of 3-hydroxyisobutyryl carnitine, which is more frequently seen in HIBCHD [29]. In this case, two variants were identified in *HIBCH*: a definitively pathogenic variant associated with a variant of unknown significance (VUS) according to ACMG criteria [32]. Based on the distinctive biochemical profile, we proposed the reclassification of the latter variant. From our perspective, basic metabolic studies are mandatory for all patients, including an L/P ratio [33], plasma amino acids and acylcarnitine profiles, and urinary organic acids, which achieved up to 80% (40/50) of the diagnostic rate, as shown in Figure 2.

The *SLC19A3* patient displayed episodic ataxia and regression after intercurrent illness (previously diagnosed with two encephalitis episodes). Neuroimaging revealed cavitations in the basal ganglia area with brain atrophy, a mandatory differential diagnosis with CI deficiency by NADH:ubiquinone oxidoreductase core subunit S1 (*NDUFS1*) and NADH:ubiquinone oxidoreductase core subunit V1 (*NDUFV1*) mutations [34], as biotin responsive basal ganglia disorders (BBGDs) are treatable. *SLC19A3* mutations were identified through a targeted nuclear gene panel, allowing personalized treatment with higher doses of thiamine and biotin with full recovery.

In non-critically ill patients, a stepwise sequential and targeted approach can be cost-effective. Nevertheless, selected cases require invasive procedures. A deep tissue biopsy can be performed ad initium for specific organ manifestation (e.g., liver disease), rapidly progressive symptoms, critically ill patients, and in cases where access to a mitochondrial/rare disorders center is delayed [1,6,35]. In the remaining cases, tissue studies prove useful for heteroplasmy confirmation or clarifying biochemical and/or genetically unclear results as functional assay proof [6,35,36]. For biochemical assays, liver, muscle, and cultivated fibroblasts cells are the main sources selected to perform tests related to mitochondrial fusion/fission defects, and OXPHOS deficiency, and mitochondrial respiratory chain enzymatic activity tests.

## 4. Discussion

LSS is a common presentation of primary MDs with high clinical heterogeneity in clinical manifestation [6]. Several patients face multidisciplinary evaluations that delay diagnosis and appropriate treatment for years [5,6]. This work represents previously recommended criteria in clinical practice for diagnosing LSS to which we associated our experience accumulated over the years [21,22,25,37,38]. Although we acknowledge that our cohort of patients was not large, we also believe that the framework presented here facilitates the early diagnosis of treatable conditions.

### 4.1. Biochemical Features

Biochemical data are important in the process of LSS diagnosis. Pyruvate links glycolysis and the tricarboxylic acid cycle, presenting as acetyl-CoA, and should be included in the initial screening of suspected LSS patients. Moreover, lactate in CSF can be higher in MDs with neurological presentation [39], for which LSS is the most common form.

A previous study revealed that the L/P ratio in blood and CSF is useful for differential diagnosis between MDs and pyruvate dehydrogenase deficiency, being more accurate in patients with higher blood lactate levels [40]. In our experience, the presence of high lactate levels associated with an L/P ratio over 20 and a strong presence of ketone bodies in the urine are the first alert towards mitochondrial pathologies [41,42].

Mitochondria are also involved in amino acids synthesis and catabolism. Therefore, the evaluation of these cellular processes can provide important metabolic insights into multiple systems simultaneously. For example, alanine levels result from pyruvate transamination and reflect both lactate and pyruvate levels, and glycine is elevated in lipoic acid deficiency due to its pivotal role in the glycine cleavage system [43,44,45]. Hyperalaninemia can also be related to hyperammonemia frequently found in organic acidurias. Citrulline is another important marker, as its decreased levels are present in approximately 80% of patients carrying *MT-ATPase6* mutations [23,24]. This finding allows directed gene testing for m.8993T>G/C and m.9176T>G/C variants [25,26]. There are limitations to the usefulness of biochemical testing, and MDs diagnosis is not possible despite normal biochemical testing. OXPHOS assessment in fibroblasts is an alternative test before more tissue collection, and should be considered, especially in infants [1]. Nevertheless, it might not be informative [46,47].

Data on urinary organic acids and acylcarnitine profile are also important, as they can direct specific therapeutic interventions. High levels of methylmalonic acid (MMA) associated with tricarboxylic acids intermediates can be identified in mtDNA depletion syndromes secondary to succinate-CoA ligase ADP-forming subunit beta (*SUCLA2*) and succinate-CoA ligase GDP/ADP-forming subunit alpha (*SUCGL1*), as well as in methylmalonic acidurias [48,49]. In ethylmalonic encephalopathy, ethylmalonic acid (EMA) is found in the urinary profile associated with methylsuccinate, butyryl, isovalerylglycines, and lactic acidosis [48]. EMA may be elevated in short-chain acyl-CoA dehydrogenase deficiency (SCADD) and glutaric aciduria type II (MADD). 3-MGA is a compound found in patients with MDs such as 3-methylglutaconic aciduria and deafness, encephalopathy and “Leigh-like” (MEGDEL) syndrome, Barth syndrome, and mitochondrial CV deficiency nuclear type 2 (MC5DN2) syndromes [48,50,51]. Moreover, mitochondrial enzyme defects (HIBCHD and ECHS1D) differ from each other by the presence of high levels of 2-methyl-2,3-dihydroxybutyric acid in urine in ECHS1D and the elevation of 3-hydroxyisobutyryl carnitine in plasma observed in HIBCHD [29,30,31].

An acylcarnitine profile can be useful in newborn screening to confirm fatty oxidation disturbances and organic acidurias. Multiple carboxylase deficiency (MCD) is a severe disorder that blocks the multiple carboxylase metabolism via interference in the biotin metabolism. Usually, it is indicated by abnormal levels of propionylcarnitine (C3) and/or hydroxyisovalerylcarnitine (C5-0H) in acylcarnitine studies and carboxylase intermediate compounds in urine workups. Nevertheless, without an MCD confirmation, a CV deficiency should be suspected in children with elevated C3 or C5-OH [48]. Additionally, a newborn screening series reported abnormal C3 and/or C5-OH levels, but enzymatic and molecular studies did not confirm MCD. Two babies in this cohort displayed neurological symptoms. The following molecular studies identified *MT-ATPase6* mutations [52].

### 4.2. Molecular Studies

In our center, we experienced an 80% diagnostic rate (40/50) using mtDNA single gene sequencing by Sanger followed by targeted nuclear gene panel and then mtDNA genome by next generation sequencing (NGS). As shown in Figure 2, steps 1 and 2 were sufficient to diagnose 62% of our cohort, and after the molecular approach (3 steps), 80% of cases were clarified. Undiagnosed cases (10/50) were directed to WES analysis. Molecular analysis is challenging in MDs cases, with variable results in biochemical screening. After inconclusive initial analysis, phenotype and data re-analysis are recommended [53,54,55]. This strategy can increase the diagnostic rate by up to 26% in WES [56]. A negative molecular result does not exclude the possibility of the patient carrying a deleterious rearrangement of DNA or replacements in functionally important non-coding regions [57,58]. Maximizing analytical efforts may enhance success in WES analysis, such as including a trio-analysis, in which familial variants and more complex inheritance patterns can be clarified [59]. mtDNA sequencing of specific affected tissue [60], methylation studies [61], and short-read sequencing (SRS), particularly in neurodegenerative disorders [62,63], are other important strategies that can yield a definite diagnosis.

Soon, rapid sequencing methods, such is long-read sequencing, can stand out as an alternative in molecular diagnosis [60]. Even though NGS enables several simultaneous analyses, results might take weeks or months to be officially reported [64]. In critical cases, we should prioritize target-gene sequencing if a suspected genotype is supported by biochemical and/or imaging methods, which may save time and avoid potentially fatal outcomes.

### 4.3. Tissue Biopsy

For many years, a specific tissue analysis was the first line approach to diagnosing MDs [6]. The evolution towards NGS performed in DNA extracted from leukocytes prevented a more invasive approach in many cases [65]. In fact, when evaluating patients suspected of having LSS, careful consideration should be given to how deep tissue fragments are obtained and the risks associated with this invasive process. Therefore, specific tissue collection should be reserved for severely ill patients who are unable to seek health support in a specialized center for rare/mitochondrial disorders, patients without alternative material sources for tests, rapidly progressive cases, and cases with specific organ involvement (e.g., the liver). For cases with conflicting genetic results, deep tissue biopsy can be necessary to confirm mitochondrial dysfunction through biochemical studies (assessing oxygen consumption via microscale oxygraphy, or OXPHOS enzyme deficiency), morphologic abnormalities (e.g., mitochondrial fission/fusion defects), confirming VUS, especially mitochondrial respiratory chain complexes, and assessing heteroplasmy levels [6,35].

## 5. Conclusions

The clinical presentation of LSS includes diverse neurological and radiological features. Since its first description in 1951, several questions related to the underlying causes of the disease remain without a definitive answer. The lack of detailed diagnostic criteria has delayed appropriate interventions and optimal prognosis. The incorporation of basic assays can enhance the number of diagnosed cases, as molecular assessment is unevenly accessible across diagnostic centers. As the diagnostic framework improves, we anticipate an adjustment in the pathway towards medical interventions in centers where NGS is not yet widely available to avoid preventable consequences and increase the quality of life of affected individuals.

## 6. Future Perspectives

Growth/differentiation factor 15 (GDF-15) and fibroblast growth factor 21 (FGF-21) are cytokine stress markers of mitochondrial dysfunction and have gained attention for their potential use in the diagnosis of MDs [11]. GDF-15 is produced in response to cellular stress, playing essential roles in both regulatory stress and inflammatory response. It was also identified in the hindbrain, where its receptor GFRAL is overtly expressed, although its exact role in MDs remains elusive [66]. Most reports claim statistical significance firmly correlating it to mitochondrial translation and deletions defects [11,67]. FGF-21 is a hormone-like cytokine linked to mitochondrial myopathies, disease severity, and respiratory chain deficient fibers [68,69]. Additionally, in genetically confirmed mitochondrial hepatopathies and translation defects, a stronger correlation is seen with GDF-15 levels rather than FGF-21 levels [70]. In vanishing white matter neurodegeneration, a non-mitochondrial early degenerative disease, GDF-15 is markedly elevated in CSF [66]. These biomarkers are also identified in multiple disorders, such as cardiovascular disorders [71], type 2 diabetes [72], and pediatric headaches [73]. Therefore, a more extensive work-up validation for adoption in routine screening for MDs is required. 

## Data Availability

The original contributions presented in the study are included in the article, further inquiries can be directed to the corresponding author/s.
